# Shock Wave-Activated Silver-Loaded Biopolymer Implant Coating Eliminates *Staphylococcus epidermidis* on the Surface and in the Surrounding of Implants

**DOI:** 10.3390/pharmaceutics15122670

**Published:** 2023-11-25

**Authors:** Martin Schulze, Melanie Nonhoff, Julian Hasselmann, Manfred Fobker, Silke Niemann, Christoph Theil, Georg Gosheger, Jan Puetzler

**Affiliations:** 1 Department of General Orthopedics and Tumor Orthopedics, Muenster University Hospital, 48149 Münster, Germany; 2 Materials Engineering Laboratory, Department of Mechanical Engineering, University of Applied Sciences Muenster, 48565 Steinfurt, Germany; 3 Central Laboratory, Muenster University Hospital, Albert-Schweitzer-Campus 1, 48149 Münster, Germany; 4 Institute of Medical Microbiology, Muenster University Hospital, 48149 Münster, Germany

**Keywords:** anti-infective silver, implant coating, implant-related infection, extracorporeal shock waves, biofilm prevention

## Abstract

Bacterial biofilms on foreign surfaces are considered a primary cause of implant-related infections, which are challenging to treat. A new implant coating was developed, containing anti-infective silver within a biocompatible polymer carrier substance. In addition to its passive effect on the implant surface, highly concentrated anti-infective silver can be released as needed via the application of high-energy shock waves. This intervention could be applied transcutaneously in a clinical setting without the need for additional surgery. We investigated the inhibition of biofilm formation and the effectiveness of eradication after activation of the coating via shock waves in an in vitro biofilm model using *Staphylococcus epidermidis* RP62A. This was performed via scanning electron microscopy and quantitative microbiology. Additionally, we examined the cytotoxicity of the new coating on normal human fibroblasts and Saos-2 osteoblast-like cells, depending on the silver concentration. All studies were compared to uncoated titanium surfaces Ti6Al4V and a conventional electroplated silver coating. Cytotoxicity toward normal human fibroblasts and Saos-2 osteoblast-like cells increased with higher silver content but remained tolerable at 6%. Compared to uncoated Ti6Al4V and the electroplated silver coating, the new coating with a silver content of 4% and 6% exhibited a significant reduction in adherent bacteria by a factor of approximately 1000. This was also evident via microscopic examination of the surface morphology of the biofilms. Furthermore, following shock wave activation, no bacteria were detectable on either the implant or in the surrounding fluid after a 24 h period.

## 1. Introduction

Implant-related infections are a major concern in orthopedic surgery [1]. Megaendoprostheses bear a risk of approximately 20% of developing periprosthetic joint infection (PJI) [2,3]. This risk may exceed 50% after multiple revisions [4,5]. The majority of PJI is caused by *Staphylococcus aureus* or coagulase-negative staphylococci such as *Staphylococcus epidermidis* [6]. The elimination of all microorganisms is essential to the healing process.

In recent years, the use of hydrogels as local drug delivery systems has been explored [1,7,8,9]. Hydrogels consist of polymers such as hyaluronic acid, chitosan, gelatine, or agarose. They have the advantage of being biodegradable and easy to load with additives because of their tissue-like structure [10]. However, hydrogels often degrade within days and therefore release the anti-infectives they contain fast with a burst release profile [7,9,11,12]. Approximately 60 to 70% of all PJI cases occur within the first two years after the implantation of a prosthesis [13]. It would therefore be beneficial to achieve sustained release of antimicrobials during this period for cases with a high risk of infection.

Poly-L-lactic acid (PLLA) is known for its slower degradation compared to other biopolymers, taking over three years to be absorbed [14,15]. PLLA is a member of the polylactic acid (PLA) family of enantiomers and is characterized by biodegradability and biocompatibility. The high crystallinity of the predominantly semi-crystalline enantiomer results in a reduced degradation rate [16]. The durability of the coating should be supported by this property, which should result in continuous release kinetics.

A bacterial biofilm presents a significant challenge in combating infections. The bacteria adhere to the foreign surface, proliferate there, and eventually form a mucous layer of polysaccharides and proteins. Within this biofilm, the bacteria are protected from host immune responses and from systemically applied antibiotics. Local antimicrobial substances on the surface of the foreign body may already be able to inhibit the onset of biofilm formation [17,18]. A novel silver-loaded biopolymer coating for implants, described and tested here, aims to prevent biofilm formation and additionally treat existing biofilm and other surrounding bacteria by releasing anti-infective silver ions on demand. In a previous study, we examined the burst release of anti-infective silver ions from the novel coating activated by extracorporeally applied high-energy shock waves [19].

In this study, we assessed the performance of the biopolymer coating in two clinical scenarios: (1) the ability of the coating to inhibit initial biofilm formation and (2) the effectiveness of the extracorporeal activation of a burst release of silver ions by shock waves to treat a pre-existing biofilm. An in vitro *Staphylococcus epidermidis* biofilm model was employed for both scenarios. Additionally, the study examined the impact of silver content on the coating’s cytotoxicity toward fibroblasts and osteoblast-like cells to attain an optimal balance between cytotoxicity and anti-infective efficacy.

## 2. Materials and Methods

### 2.1. Sample Preparation

Ti6Al4V Grade 5 ELI discs were chosen as substrate material, given their frequent use in orthopedics as a biomaterial [20]. The geometry was designed with a 14 mm outer diameter, 1.5 mm thickness, and a 2 mm centric hole. Table 1 shows the number of samples per coating and test.

The Ti6Al4V samples used for coating underwent initial sterilization via immersion in 70% ethanol. All subsequent steps were performed using an S2 workbench. The surface of the discs was prepared by etching in 0.5 M oxalic acid (6.3 g/100 mL H_2_O) at 100 °C for 45 min, creating a finely structured topography in order to enhance the adhesion of the coating. After cooling to room temperature, the discs were rinsed with 0.5 M calcium lactate solution.

The coating was prepared via manual dip-coating in a 10% solution of poly-L-lactide (PLLA, RESOMER L 206 S, Evonik Health Care, Darmstadt, Germany) and chloroform. Subsequently, 25 µL of silver ions at different concentrations (2, 4, and 6%) were added to 1 mL of this solution. After immersion, the discs were air dried until the chloroform had evaporated completely, resulting in a coating thickness of approximately 10 µm.

Electroplated silver samples with identical geometry as the Ti6Al4V samples were provided by implantcast GmbH (Buxtehude, Germany).

### 2.2. Cytotoxicity

The cytotoxicity of the different coating variants was measured via WST-1 assay with normal human fibroblasts (NHF; PromoCell GmbH, Heidelberg, Germany) and Saos-2 osteoblast-like cells (DSMZ, Braunschweig, Germany). Cells were cultured in Dulbecco’s modified Eagle’s medium (DMEM) with 10% fetal calf serum (FCS), 2 mM L-glutamine, and 1% antibiotic-antimycotic in 12-well plates at 37 °C and 5% CO_2_.

NHF cells were seeded onto the samples at a concentration of 0.35 × 10^5^ cells per well in a 12-well plate, while Saos-2 cells were seeded onto the samples at a concentration of 0.4 × 10^5^ cells per well in another 12-well plate. The medium was changed to 1100 µL DMEM with 5% FCS and 120 µL WST-1 for the assay. The resulting optical density (OD) of the formazan dye was measured at a wavelength of 450 nm using a spectrophotometer (BMG Labtech FLUOstar Optima Fluorimeter, Ortenberg, Germany) at different time points for 24 h.

The percentage of cell viability was calculated from the measured optical density with the reference of the cell control by (OD_sample_/OD_control_) × 100.

### 2.3. Biofilm Assay

To observe the bacterial growth on the surface of the samples, *Staphylococcus epidermidis* RP62A (ATCC-35984; American Type Culture Collection, Manassas, VA, USA) was cultured overnight in Tryptic Soy Broth (TSB). The culture was diluted 1:200 in TSB plus 0.25% glucose, leading to an initial bacterial load of around 1 × 10^5^ CFU/mL. The sterile samples were placed in a 12-well plate, and 1 mL of the inoculum was added. The plate was then incubated at 37 °C for 24 h. The next day, the samples were transferred to another sterile container and washed with phosphate-buffered saline (PBS). The discs were then sonicated with 1 mL of PBS for 5 min to remove the biofilm. To determine the number of CFU, serial dilutions of the resulting supernatant were plated on Columbia blood agar and incubated overnight at 37 °C. After 24 h, colonies were assessed using a colony counter (Biocount 5000, Biosys GmbH, Karben, Germany). The minimum detectable level was 10 CFU/mL.

### 2.4. SEM Analysis

For qualitative analysis of the sample surface after biofilm growth, we utilized the scanning electron microscope (SEM; Zeiss EVO MA10, Carl Zeiss Microscopy GmbH, Jena, Germany). Prior to analysis, the samples were fixed with an ethanol (EtOH) series that increased in concentration (30 min with 70% EtOH, followed by 15 min each with 80%, 90%, 96%, and 100% EtOH). After fixation, the samples were mounted onto aluminum stubs using carbon adhesive discs. To ensure electrical conductivity, a thin film of gold was deposited on the surface by DC sputter coating (Polaron E5000, Polaron Equipment Ltd., Watford, UK) for 45 s at a current of 10 mA. The images were obtained using an acceleration voltage of EV = 5 kV, a working distance of WD = 13 mm, and a magnification of 5000× in high vacuum mode.

### 2.5. Shock Wave Application after Biofilm Growth

To assess the anti-infective effect of the coating’s activation by extracorporeally applied shock waves on a pre-existing biofilm, firstly, a biofilm was grown on both PLLA + 6% Ag and uncoated discs as described above. Subsequently, the discs were transferred to sterile bags (SteriBags, Bürkle GmbH, Bad Bellingen, Germany) filled with 1 mL of PBS. The bags were positioned in the shock wave setup as described earlier [21] and exposed to 1000 pulses per quadrant on one side. The energy flux density was set to 1.24 mJ/mm^2^, and the frequency was 3 Hz, which resulted in a total energy release per sample of 40.55 J. The test setup was filled with water as a transfer medium for the shock waves due to its comparable acoustic properties to soft tissue. Specimens of the control groups were positioned in the setup but not exposed to shock waves.

Half of the samples and supernatant, e.g., PBS inside the SteriBags after shock wave application, were transferred to a new 12-well plate and incubated for another 24 h at 37 °C. The other half was analyzed immediately. In order to analyze the biofilm, discs were sonicated in 1 mL of PBS for 5 min, whereas for planktonic bacteria, the supernatant from the SteriBags was collected. The number of viable bacteria was assessed by plate counting, as already described above.

### 2.6. Statistical Analysis

GraphPad Prism 10 (GraphPad Software, Inc., Boston, MA, USA) was used for statistical analysis and plotting of the results. The results are expressed as mean ± standard deviation or median ± interquartile range of at least 3 independent experiments. All results were tested for normal distribution using the Kolmogorov–Smirnov test. WST-1 assays were examined using a repeated measures one-way ANOVA and Bonferroni post-tests. CFU data from the biofilm assay were analyzed using one-way ANOVA and Bonferroni post-tests, while the shock wave on biofilm assay was interpreted with two-way ANOVA and Bonferroni post-tests. A *p*-value was considered significant if *p* < 0.05 (*), very significant if *p* < 0.01 (**), and highly significant if *p* < 0.001 (***).

## 3. Results

### 3.1. Cytotoxicity

**Figure 1:**
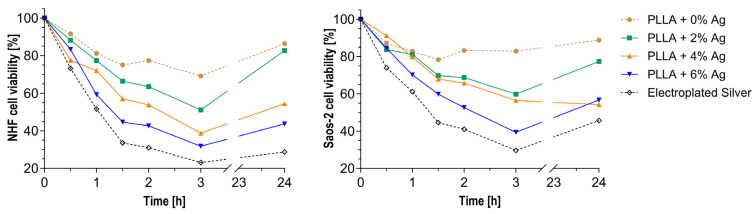
Viability of NHF (**left**) and Saos-2 cells (**right**) was tested in a WST-1 assay with various silver concentrations in the PLLA coating, using electroplated silver as a control. The cell viability in reference to the cell controls increased over 24 h. All viabilities of the PLLA and silver samples exceed those of the electroplated silver samples.

A WST-1 assay was conducted to evaluate the cytotoxic impact of the coatings as a function of the silver content (Figure 1). The optical measurement of formazan produced by viable cells was used to calculate the percentage of cell viability relative to the cell control. While the viability of both types of cells initially decreases, it significantly improves within 24 h. The overall viability is inversely proportional to the amount of silver mixed within the coating. However, the values of the new coating exceed those of the clinically used electroplated silver coating.

### 3.2. Biofilm Assay

**Figure 2:**
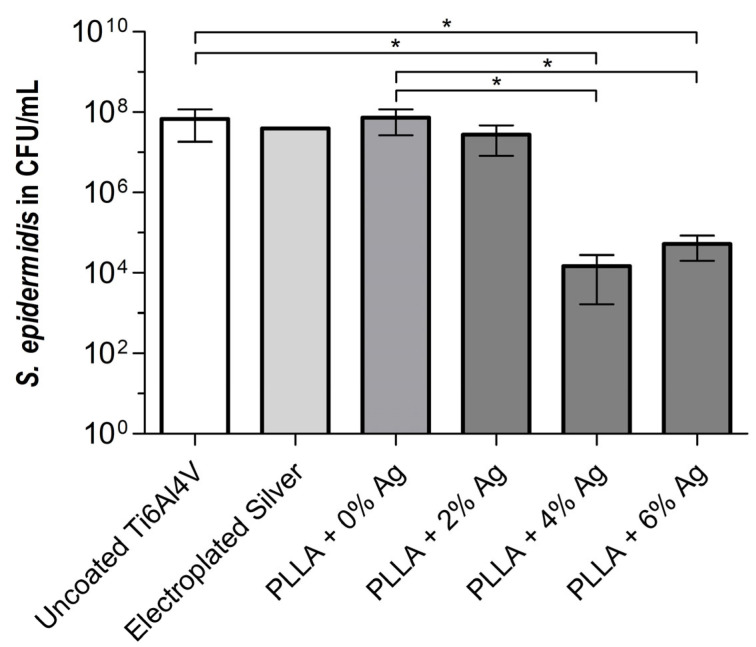
Biofilm formation of *S. epidermidis* on Ti6Al4V discs with different coatings. The PLLA coatings containing 4% and 6% silver show significantly less bacterial growth than the uncoated sample and the pure PLLA coating (*p* < 0.05). Bars indicate mean; error bars indicate standard deviation; * *p*-values < 0.05 from one-way ANOVA.

The anti-infective effect on bacteria was analyzed in a biofilm assay for 24 h using *S. epidermidis* RP62A (Figure 2). All three controls (uncoated Ti6Al4V, electroplated silver, and PLLA without silver) exhibited strong bacterial colonization in the range of 10^7^ to 10^8^ CFU/mL. No significant reduction in bacterial growth was observed on the PLLA coating containing 2% silver. However, compared to the uncoated control and the PLLA-only control, the PLLA coatings containing 4% and 6% silver demonstrated a significant reduction in the bacterial load with an approximately 1000-fold decrease (*p* < 0.05). There is a slight increase in the bacterial load at 6% silver in comparison to that at 4% silver.

**Figure 3:**
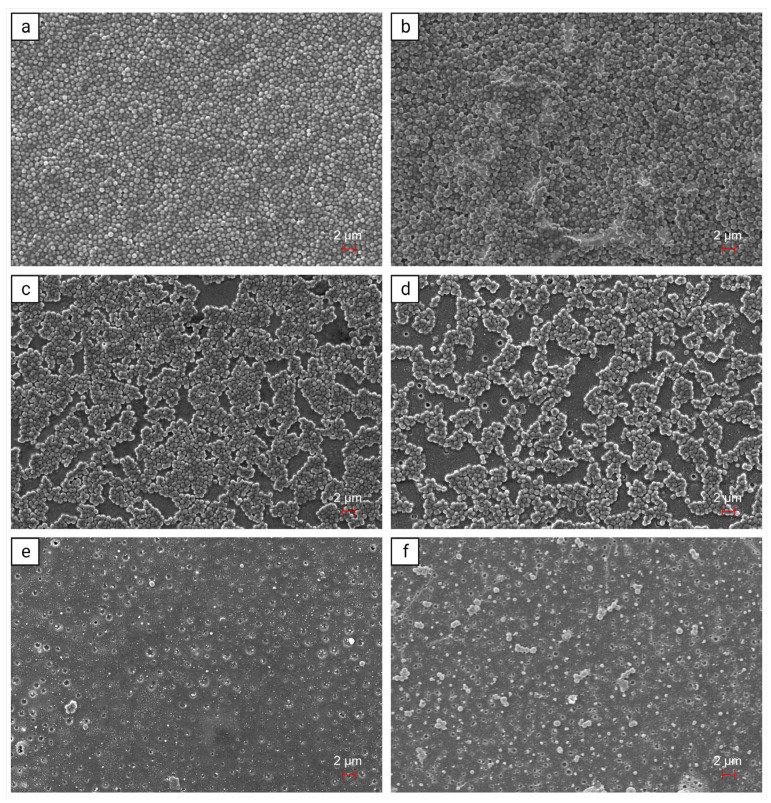
Scanning electron microscope (SEM) images illustrate *S. epidermidis* biofilm on sample surfaces after 24 h of incubation at 5000× magnification. The samples depicted include (**a**) Ti6Al4V, (**b**) electroplated silver, (**c**) PLLA + 0% Ag, (**d**) PLLA + 2% Ag, (**e**) PLLA + 4% Ag, and (**f**) PLLA + 6% Ag.

In addition, the bacterial growth was qualitatively analyzed using SEM images. The sample without coating (Figure 3a) and the conventional electroplated silver sample (Figure 3b) were both completely covered in thick layers of Staphylococci. However, a slight reduction is noticeable for the electroplated silver sample. In the coatings that contain pure PLLA (Figure 3c) and 2% silver (Figure 3d), gaps in the bacterial lawn were observed, partly exposing the implant surface. With 4% (Figure 3e) and 6% silver (Figure 3f), only very few staphylococci were visible. The SEM analysis confirms the substantial decrease in bacterial growth when silver concentrations reach 4% or higher. These findings align with those of the prior quantitative microbiological analysis.

### 3.3. Shock Wave Application after Biofilm Growth

**Figure 4:**
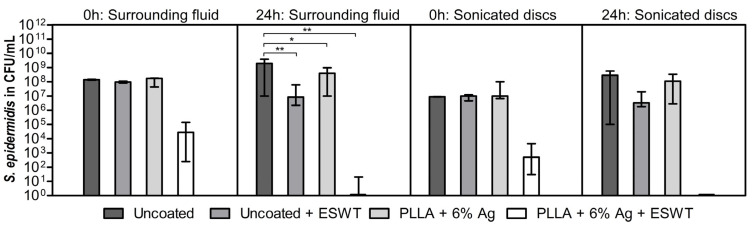
Planktonic bacteria in the surrounding fluid and sessile *S. epidermidis* RP62A on uncoated and PLLA + 6% Ag coated titanium discs after 0 h and 24 h with and without shock wave application. The silver coating with applied shock waves shows lower bacterial load than all control groups. Bars indicate mean; error bars indicate standard deviation; ** *p*-value < 0.01 and * *p*-value < 0.05 from two-way ANOVA.

Since previous investigations indicated that the highest antibacterial effect, including acceptable cytotoxicity, is expected with a silver content of 6%, the experiments for shock wave activation in a scenario with a pre-existing biofilm were conducted using the 6% silver coating. Figure 4 illustrates the effect of additional shock waves after biofilm growth on the samples. In contrast to the coated samples, the uncoated control specimens displayed a significant presence of planktonic bacteria within the surrounding fluid and exhibited a robust biofilm formation. Although there was a significant decrease in the number of bacteria in the surrounding fluid in all three other groups when compared to the uncoated control without shock waves (*p* < 0.01 and *p* < 0.05), only the combination of shock waves and silver in the coating resulted in the eradication. In the shock wave groups, the presence of 6% silver in the PLLA coating led to a highly significant decrease overall compared to the uncoated samples. After a 24 h incubation following the shock wave application, no viable bacteria could be cultured from the surrounding fluid and from the sample surface, indicating eradication of planktonic and sessile *S. epidermidis.*

## 4. Discussion

The present study examined the anti-infective effect of a biopolymer implant coating containing silver ions in two clinically relevant scenarios: First, a biofilm assay was used to show that the coating with 4 and 6% silver content is able to significantly reduce initial biofilm formation. Previous research disclosed that extracorporeal activation of a burst release of the silver content by means of a shock wave has a high anti-infective effect [22]. Secondly, this has now been demonstrated in the case of a pre-grown biofilm of *Staphylococcus epidermidis* on the sample surface when coated with 6% silver ions. Furthermore, all silver concentrations in the PLLA coating exhibited satisfactory cytotoxicity.

### 4.1. Cytotoxicity

A WST-1 assay was utilized to assess the cytotoxicity of the coating on NHF and Saos-2 cells. In general, this assay demonstrated a higher cell proliferation for the biopolymer coating containing silver ions than the electroplated silver coating, which has been in clinical use for several years in order to prevent periprosthetic infections in both cell types, even at the highest silver concentration tested (6%).

The cytotoxic impact of silver is generally recognized, with levels of over 300 ppb in the bloodstream being toxic [23]. Argyria, leukopenia, and damage to the kidneys, liver, and neural tissue are all likely to result from excessive levels of silver in the bloodstream [24,25,26]. However, studies on the preclinical and clinical use of electroplated silver megaprostheses, which are commonly used today, have not revealed any adverse side effects of silver [27,28,29,30]. Since the viability of NHF and Saos-2 cells in all samples with this novel coating and at all silver concentrations used is greater than that in the galvanic silver samples, no systemic side effects are anticipated, and an enhancement in local tolerance is expected.

In previous cases, it has been reported that antibacterial surface modifications of implants may inhibit osseointegration as the antibacterial properties could also hinder cell adhesion [12,31,32]. The coating’s intended use is non-articulating surfaces without direct or extensive bone contact. Although osteoblast-like Saos-2 cells could grow on the coating, this specification is reasonable because the activatable properties cannot be utilized on surfaces that are covered by bone. This is because the activation of the silver burst release by disruption of the coating would not be possible, as shock waves cannot be transferred efficiently through bone due to its significantly higher impedance compared to soft tissue. In general, it is crucial to achieve a balance between the cytotoxic effect and the anti-infective effect.

### 4.2. Biofilm Assay

To assess the influence on biofilm growth, a biofilm assay was conducted using *S. epidermidis* RP62A. All samples underwent thorough washing to ensure that only the sessile bacteria of the biofilm were present. A significant decrease in sessile bacteria in the biofilm was observed with silver concentrations of 4% and 6% in the coating, respectively. This is consistent with the observation of only sporadically visible Staphylococci in the SEM images. In addition, some of the bacteria in the SEM images showed a partially angular structure. This could be due to too rough dehydration, or it could mean that these bacteria were no longer viable. Since the bacteria in the other images look vital (circular appearance) and the same dehydration technique was used, it can be assumed that a large proportion of the bacteria in the 4% and 6% silver samples were already dead before the SEM analysis. In the SEM images, we observed that higher concentrations of silver in the coating had a more potent anti-infective effect. However, the CFU count of 6% silver was slightly higher compared to 4%, although not significant. We attribute this result to the manual process involved in manufacturing the coating that might not yet achieve a perfectly homogenous distribution of the coating on the surface. Since the error is visible in both the quantitative microbiological analysis and the qualitative SEM images, we cannot assume that the error was caused by this methodology.

*Staphylococcus aureus* and *Staphylococcus epidermidis* commonly cause implant-related infections, and a growing resistance to antibiotics is reported, potentially complicating antimicrobial treatment [33,34]. Therefore, non-antibiotic antimicrobials like silver ought to be used, preferably utilizing the anti-infective mechanism to combat infections. Silver ions serve a dual role in inhibiting and killing bacteria. Initially, silver ions adhere to the murein wall, modifying its permeability as a bacteriostatic effect. Subsequently, the ions bind to thiol groups in enzymes, rendering them nonfunctional. As a consequence, the tricarboxylic acid cycle and respiratory chain are disrupted, leading to the accumulation of hydroxyl radicals. These radicals are known to harm bacterial DNA and have a bactericidal effect [35,36]. The bactericidal effect is necessary to prevent the development of resistance. Surviving bacteria remain exposed to the active ingredient (in this case, silver), increasing the likelihood of resistance. Aiming for the highest tolerable concentration is desirable to achieve not only a bacteriostatic but also a bactericidal effect.

Given that PLLA degrades slowly due to its highly crystalline nature, achieving the highest attainable concentration of silver, evenly distributed throughout the material, would ensure a continuous release of bactericidal levels of silver. High silver concentrations are required as physiological fluids can reduce the effect of silver [37,38]. For this reason, we presume that the 6% silver in the coating likely represents the lower limit of what is possible. Future experiments may reveal that this concentration should be increased further in a physiological environment.

### 4.3. Shock Wave Application after Biofilm Growth

The primary objective of the coating is to prevent the formation of bacterial biofilms on the implant. In cases of a high bacterial load, such as during acute infection, the additional release of silver via high-energy shock waves can lead to a significant reduction or even eradication in this model. Therefore, on-demand activation could be an additional modality in addressing implant-related infections. Previous studies have demonstrated a reduction in biofilm presence both in vitro and in vivo when exposed to shock wave treatment [39,40,41,42,43]. The concrete effects of the shock waves have not yet been fully understood. Possible outcomes include direct bacteria elimination via wave impact or disruption of the biofilm, leading to bacterial return to a planktonic state. The latter is considered more plausible and results in increased accessibility of bacteria to anti-infective agents [40].

A previous study demonstrated that silver was effectively released from the disrupted PLLA layer, exceeding the MIC for *S. epidermidis* [19]. The amount of released silver in the surrounding fluid was effective against *S. aureus*, *S. epidermidis*, and *E. coli* [19].

To investigate the synergistic effect of the direct impact of shock waves on the biofilm and the anti-infective action of the released silver within a single model, shock waves were applied directly to a biofilm that had been cultivated on the 6% silver-containing coating for 24 h. When the coating was activated by shock waves, bacteria on the implant surface were successfully eradicated after 24 h. Although the bacterial load in the uncoated samples with shock waves decreased slightly compared to the uncoated samples without shock waves, the decrease was not notable compared to the silver and shock wave groups. This supports the hypothesis that shock waves alone do not effectively eliminate bacteria, whereas the PLLA + 6% Ag coating shows a strong anti-infective effect when activated via shock waves due to the release of silver ions.

In a clinical setting, this activation is expected to be used as a non-invasive therapy when late infection occurs. The biopolymer PLLA, with a degradation period exceeding three years, acts as a silver reservoir in the coating, which is released by the shock waves [14,15,19]. The shock wave device has already demonstrated efficacy in orthopedic clinics for various therapies, including treating plantar fasciitis and epicondylitis [44,45]. Shock waves have been reported as safe multiple times, and there is evidence that they positively impact fracture healing [45,46,47]. In various in vitro and in vivo experiments, evidence suggests that shock wave therapy induces angiogenesis via the release of nitric oxide and subsequent cascade [48]. Additionally, there is an observed upregulation of TGF-β1 stimulating the proliferation and differentiation of fibroblasts [48].

### 4.4. Limitations

There might be some variability in the coating due to the present manual dipping manufacturing process. This variation might account for the findings presented in Figure 2 with regard to the 4% and 6% coating. The development of a standardized manufacturing process is currently ongoing.

The pathogen *S. epidermidis* was selected for testing, given its high clinical incidence and propensity to form antibiotic-resistant biofilms [49]. However, it is important to note that various other microorganisms, such as *Staphylococcus aureus*, *Escherichia coli*, and others, should also be tested with this coating.

In the clinical application of the coating, additional environmental influences will come into play that have not yet been included in the in vitro tests. The outcomes of the anti-infective effect will be affected by the immune system, and physiological fluids also have properties that affect the effectiveness of silver [37,38]. It is advisable to conduct future in vitro trials that closely imitate these circumstances, in addition to carrying out in vivo trials to evaluate the impact.

Additionally, the studies to date have been carried out within a few days, so the duration of the treatment is relatively short. The aging and potential degradation of the coating in the body is a critical aspect of its efficacy. Therefore, prospective future trials ought to be carried out to examine this aspect, either via artificial aging of the coating or via the long-term verification of its effectiveness.

## 5. Conclusions

This novel PLLA coating infused with silver ions exerts preventive and interventional effects against implant-related infections.

The preventive effect was demonstrated by both qualitative and quantitative reductions in biofilm on the implant surface. Acceptable cytotoxicity with sustained fibroblast and osteoblast proliferation was achieved.

The interventional effect was demonstrated by activating the coating with shock waves on cultured biofilm, resulting in eradication within 24 h.

The 6% silver concentration was selected for further studies because this concentration in vitro achieves an adequate balance between acceptable cytotoxicity and good antibacterial effectiveness. This coating could serve as a significant advantage in the clinical treatment of periprosthetic infections. 

## 6. Patents

A patent application has been filed for the coating (international publication number: WO2023025944).

## Figures and Tables

**Table 1:** Matrix of samples used per test.

Coating	WST-1 NHF	WST-1 Saos-2	Biofilm	Biofilm (with/without Shock Wave)
Ti6Al4V	-	-	12	6/4
Electroplated Silver	1	1	1	–
PLLA + 0% Ag	6	6	6	–
PLLA + 2% Ag	6	6	3	–
PLLA + 4% Ag	6	6	6	–
PLLA + 6% Ag	6	6	5	6/6

NHF: normal human fibroblasts; Saos-2: osteoblast-like cells.

## Data Availability

The data presented in this study are available upon request from the corresponding author. The data are not publicly available due to privacy restrictions.
